# Assessment of Relative Contributions of Lifestyle, Behavioral and Biological Risk Factors for Cervical Human Papillomavirus Infections in Female Sex Workers

**DOI:** 10.3390/v17040485

**Published:** 2025-03-28

**Authors:** Imran Morhason-Bello, Kyeezu Kim, Yusuf Bello, Yinan Zheng, Sunday Oyerinde, Oluwasegun Caleb Idowu, Miquel Ángel Pavón, Kathy Baisley, Jun Wang, Adeola Fowotade, Mamoudou Maiga, Musa Jonah, Elizabeth Nicole Christian, Olufemi Ogunbiyi, Isaac Adewole, Lifang Hou, Suzanna C. Francis, Deborah Watson-Jones

**Affiliations:** 1 Obstetrics and Gynaecology Department, Faculty of Clinical Sciences, College of Medicine, University of Ibadan/University College Hospital, Ibadan 200285, Nigeria; sunday.oyerindedimeji@gmail.com (S.O.); idoscale@gmail.com (O.C.I.); 2 Institute for Advanced Medical Research and Training, College of Medicine, University of Ibadan, Ibadan 200285, Nigeria; belloyusufolatunji17@gmail.com; 3 HPV Consortium, College of Medicine, University of Ibadan, Ibadan 200285, Nigeria; temilabike@gmail.com (A.F.); fogunbiyi@gmail.com (O.O.); ifadewole@gmail.com (I.A.); 4 Department of Social and Preventive Medicine, Sungkyunkwan University School of Medicine, Suwon-si 16419, Republic of Korea; kyeezu.kim@skku.edu; 5 Department of Preventive Medicine, Northwestern University Feinberg School of Medicine, Chicago, IL 60611, USA; y-zheng@northwestern.edu (Y.Z.); j-wang4@northwestern.edu (J.W.); mamoudou.maiga@northwestern.edu (M.M.); l-hou@northwestern.edu (L.H.); 6 Robert J. Havey, MD Institute for Global Health, Northwestern University Feinberg School of Medicine, Chicago, IL 60611, USA; elizabeth.christian@northwestern.edu; 7 Infection and Cancer Laboratory, Cancer Epidemiology Research Program, ICO, Bellvitge Biomedical Research Institute (IDIBELL), Centro de Investigación Biomédica en Red de Epidemiología y Salud Pública (CIBERESP), 08908 Barcelona, Spain; mpavon@iconcologia.net; 8 Department of Infectious Disease Epidemiology, Faculty of Epidemiology and Population Health, London School of Hygiene and Tropical Medicine, London WC1E 7HT, UK; kathy.baisley@lshtm.ac.uk; 9 Department of Medical Microbiology, College of Medicine, University of Ibadan, Ibadan 200285, Nigeria; 10 Department of Obstetrics and Gynecology, Faculty of Clinical Sciences, College of Health Sciences, University of Jos, Jos 930105, Nigeria; drmusaj@yahoo.com; 11 Department of Pathology, College of Medicine, University of Ibadan, Ibadan 200285, Nigeria; 12 International Statistics and Epidemiology Group, London School of Hygiene and Tropical Medicine, London WC1E 7HT, UK; suzannacf@gmail.com; 13 Clinical Research Department, Faculty of Infectious and Tropical Diseases, London School of Hygiene and Tropical Medicine, London WC1E 7HT, UK; deborah.watson-jones@lshtm.ac.uk; 14 Mwanza Intervention Trials Unit, National Institute for Medical Research, Mwanza P.O. Box 1462, Tanzania

**Keywords:** female sex workers, HPV, cervical, oral, anal, sexual debut, concordance

## Abstract

This study aimed to identify and quantify the relative and collective contributions of lifestyle, behavioral, and biological risk factors to cervical HPV infections among female sex workers (FSWs) in Ibadan, Nigeria. This cross-sectional study was part of the Sexual Behavior and HPV Infections in Nigerians in Ibadan project and involved 182 FSWs for whom complete data on HPV genotypes were available. Quantile-based g-computation was employed to assess the relative and collective contributions of risk factors to any cervical HPV/hrHPV infections and multiple cervical HPV/hrHPV. The collective contribution of all selected risk factors to multiple high-risk cervical HPV was 2.47 (95% CI: 0.97–3.23). The number of other anatomic sites with HPV infections showed the highest positive relative contribution to multiple cervical HPV/hrHPV. Alcohol consumption and the total number of sexual partners contributed to high-risk cervical HPV and multiple cervical HPV/hrHPV, while age at first vaginal sex had a negative relative contribution. This study highlights the significant contribution of HPV infections in multiple anatomic sites as a risk to the acquisition of cervical HPV in FSWs. Routine screening protocols should be enhanced to include multiple anatomic sites, and targeted educational programs are recommended to address the specific risks faced by FSWs.

## 1. Introduction

Cervical cancer (CC) is the fourth leading cause of cancer-related deaths among women globally. In 2022, there were 662,301 new cases of CC worldwide and about 348,874 deaths from the disease [1]. In Nigeria, CC is the second most common cancer after breast cancer [2] and is also the most common reproductive tract cancer in women. In Nigeria, there are over 12,000 new cases and nearly 8000 deaths of CC each year, which account for almost half of its burden in West Africa [3].

Most Human Papillomavirus (HPV) infections are sexually transmitted following unprotected sexual behaviors, and in most cases, nearly up to 90% of these infections are cleared within two years of acquisition. The persistence of high-risk Human Papillomavirus (hrHPV) infection is responsible for nearly 100% of CC [4,5]. Of all the 13 known hrHPV infections, nearly 70% of CC is associated with HPV-16 and 18 genotypes [6,7]. The acquisition and transmission of hrHPV can be primarily prevented by HPV vaccination and sexual education, screening of sexually active women for HPV infections and precancers, and detection of early invasive cancer for definitive treatment [8].

Several studies and reviews have identified the risk factors associated with cervical HPV infections. Identified risk factors include Human Immunodeficiency Virus (HIV) infection, smoking, alcohol use, and young age at first pregnancy [9]. Risk factors related to sexual behavior include multiple sexual partners, young age at first sexual intercourse, high-risk sexual partner(s), and sexual contact with HPV-infected individuals, among others [10]. Similar findings have been reported across sub-Saharan Africa, with multiple sexual partners and age at first sexual intercourse being the strongest contributing risk factors [11]. These risk factors may be complementary rather than independent; for example, the consumption of alcohol is believed to increase the risk of HPV infection by its association with risky sexual behaviors [12]. There are limited data on the relative contribution of each of the identified risk factors as well as the relationship between these risk factors.

Female sex workers (FSWs) are particularly at high risk for cervical hrHPV infections and CC because they engage in more sexual risk behaviors relative to women in the general population [13]. Previous studies showed that FSWs are two-times more likely to acquire cervical HPV infections compared to women in the general population [14,15]. This increased risk among FSWs may be attributed to factors, such as frequent partner changes, multiple partners, unprotected sexual practices, smoking, use of oral contraceptives, early onset of sexual activity, a higher incidence of Sexually Transmitted Infections (STIs), and concurrent HIV infection.

Understanding the relative and collective contributions of these risk factors for high-risk cervical HPV is crucial for developing targeted public health interventions aimed at preventing CC, particularly in high-risk populations such as FSWs who are more vulnerable. Therefore, this study aims to identify and quantify the contributions of lifestyle, behavioral, and biological risk factors to cervical HPV infection among FSWs in Ibadan, Nigeria, and provide recommendations for tailored health promotion strategies to reduce the burden of CC among FSWs in Nigeria. By addressing this objective, this research seeks to contribute to the broader goal of enhancing preventive measures and reducing the risk of high-risk cervical HPV infections, a known percussor for CC.

## 2. Materials and Methods

### 2.1. Study Design, Population, and Study Site

As part of the Sexual Behavior and HPV Infections in Nigerians in Ibadan (SHINI) project conducted in Ibadan, Nigeria, this cross-sectional study focused on FSWs [16]. Brothels were selected from the six urban Local Government Areas (LGAs) in Ibadan: Ibadan North, Northeast, Northwest, Southwest, Southeast, and Akinyele.

### 2.2. Study Procedures

#### 2.2.1. Sampling and Enrolment of Study Participants

Trained field workers mapped the list of brothels in the selected LGAs prior to participants’ enrolment. Female research assistants interviewed the manager or the head of the FSWs and then compiled a list of eligible participants, assigning each a unique number. In brothels with 10 or fewer FSWs, all eligible participants were invited. In brothels with 11 or more eligible FSWs, 90% were selected through simple random sampling. Research assistants visited each brothel, distributed information pamphlets, and provided group and individual explanations of the study’s objectives in the participants’ rooms. Following this, signed consent was obtained, including consent for the storage of samples for future use.

#### 2.2.2. Interview, Clinical Examination, and Sample Collection

Interviews and sample collection took place in each participant’s bedroom. Female research assistants conducted face-to-face interviews using questionnaires in English or pidgin English for those who did not fully understand English fluently. The interview included socio-demographics, sexual behaviors, lifestyles such as alcohol consumption and smoking, STI history, and awareness of HPV and its vaccination. Following the interview, a female nurse collected blood samples and performed rapid diagnostic HIV testing (RDT) using the Nigerian HIV test protocol [17]. HIV-positive participants were referred to government-designated HIV care centers for verification and treatment. Anonymous RDTs were provided for those who preferred not to disclose their HIV status.

An experienced nurse collected biological samples from the cervix, vulva, oral and anal cavities, and placed each sample in separate vials. For the oral sample, participants used 10 milliliters of Procter & Gamble’s Scope mouthwash to gargle and rinse their mouths for 30 s. Each participant observed the nurse performing the gargle and rinse procedure. After collecting the sample in a labelled 10 mL bottle, the samples were immediately stored in a cold box at 2–4 °C. For the vulvar sample, the tip of a Dacron swab was used to gently rub the area around the vaginal opening, avoiding contact with the urethral aperture. To obtain a cervical sample, a sterile Cusco speculum was inserted into the vagina to expose the cervix. A new Dacron swab was then gently rotated 360 degrees within the cervical opening to avoid damage and potential bleeding. The participant was positioned on their left side for the anal sample collection. A Dacron swab was carefully inserted and rotated 360 degrees along the anal verge, extending approximately 5–6 cm beyond the anal edge. Each swab was placed in a labelled and barcoded 2 mL cryotube, which was then stored in a cold box at 2–4 °C. All samples were stored in a −80 °C freezer at the Institute of Advanced Medical Research and Training, University of Ibadan, Nigeria. Each participant received an incentive as a token of appreciation.

#### 2.2.3. HPV Genotyping

DNA was extracted from the mouthwash using the Maxwell 16 LEV Blood DNA Kit (Promega Corp., Madison, WI, USA). For the dry swabs collected from the cervical, vulvar, and anal regions, the Maxwell 16 Buccal Swab LEV DNA Purification Kit was used, following the previously outlined protocols [16].

The AnyplexTM II HPV28 (Seegene, Seoul, South Korea) assay was employed at the Catalan Institute of Oncology in Spain for HPV genotyping. Ten microliters of sample DNA was used for the AnyplexTM II HPV28 detection test, following the manufacturer’s instructions [18]. This assay detects 28 HPV genotypes, including 13 hrHPV (HPV-16, -18, -31, -33, -35, -39, -45, -51, -52, -56, -58, -59 and -68), lrHPV (HPV-6, -11, -40, -42, -43, -44, -53, -54 and -70) and possibly carcinogenic genotypes (HPV-26, -61, -66, -69, -73 and -82). Human beta (β)-globin served as the internal control (IC) in each sample, enabling differentiation between valid and invalid HPV results. Samples were categorized as invalid if they lacked amplification of the IC, despite being HPV negative. HPV-negative samples with a positive IC result and HPV-positive samples were classified as valid.

### 2.3. Data Management and Statistical Analysis

Double entries of the data were managed using the REDCap program (Vanderbilt University, Nashville, TN, USA). The raw data were stored and exported in CSV format. The Quantile-based G-Computation (QGC) analysis was performed using R 4.2.

Descriptive statistics for selected variables were summarized using frequency and percentage for categorical variables and means and standard deviations for continuous variables. The primary outcomes were any HPV infection, any of the 13 hrHPV infections, multiple HPV infections, and multiple hrHPV infections in the cervix. HPV prevalence was categorized according to the 2009 IARC epidemiological oncogenic classification. Independent variables included age, ethnicity, Body Mass Index (BMI), weekly income from sex work, duration of engagement in commercial sex activities, years of formal education completed, number of vaginal sex partners throughout life, age at first vaginal sex, age of first vaginal sex partner, number of weekly transactional sex partners, alcohol consumption, and the number of other sites with HPV infection aside from the cervix.

The Spearman correlation coefficient was used to examine the relationship between selected risk factors for cervical HPV infection. QGC was employed to assess both the relative and collective contributions of nine risk factors (income from sex work, duration of sex work, years of education, total number of partners, age of first vaginal sex, number of weekly transactional sex, alcohol consumption, and number of other HPV sites besides cervix) to each outcome variable, with age, ethnicity, and BMI included as covariates. In QGC, the weight represents the percentage of the effect of each risk factor with the same direction, capturing its relative contribution. A larger absolute value of the weight in the relative contribution indicates a greater contribution; positive values signify a factor’s contribution to a positive association between the combined risk factors and the outcome, while negative values represent a contribution to a negative association. The collective (overall) association, represented by β reflects the change in the outcome variable per quartile difference in the combined risk factors. All statistical analyses were performed at a 0.05 level of significance.

## 3. Results

Of the 315 FSWs who participated in this study, 182 provided complete data on HPV genotypes as well as lifestyle, behavioral, and biological factors. The mean age of the FSWs was 30.9 ± 6.4 years. The majority of FSWs were Igbo (31.3%), followed by Yoruba (7.7%). The average age of sexual debut among FSWs was 17.5 ± 2.7 years, and the average weekly income was NGN 10,560.4 ± 7295.4. FSWs had HPV infection in at least two other anatomic sites in addition to the cervix (Table 1).

**Figure 1:**
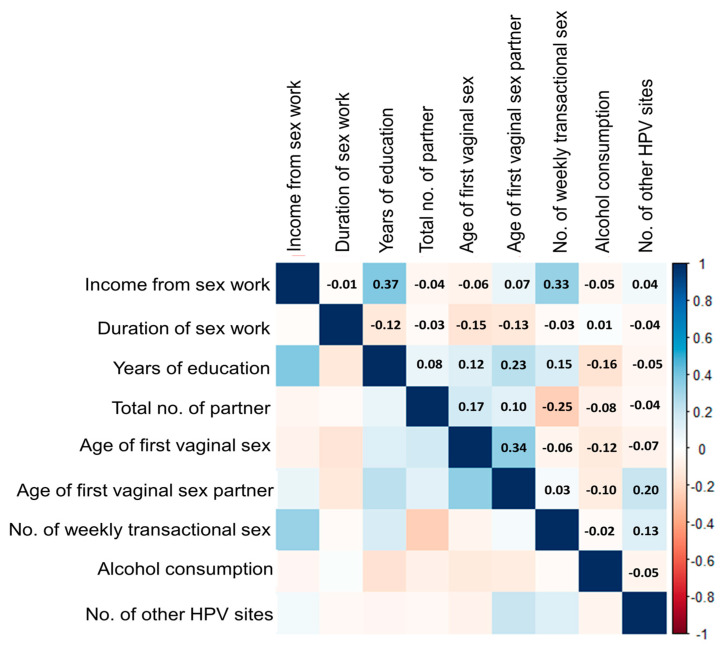
Spearman correlations between the selected risk factors and cervical HPV.

Figure 1 shows the Spearman correlations between the selected continuous risk factors. Although the associations were generally weak, the strongest positive relationship among the risk factors for cervical HPV was between years of education and weekly income from sex work (R = 0.37), followed by the relationship between age at first vaginal sex and partner’s age at first vaginal sex (R = 0.34) and the correlation between the amount of weekly transactional sex and income from sex work (R = 0.33). There was a negative relationship between the total number of partners and the amount of weekly transactional sex. Other relationships among the selected risk factors showed almost none to very weak correlations. The corresponding *p*-values of the Spearman correlations are provided in Supplementary Table S1.

Table 2 shows the relative and collective contributions of each selected risk factor to any cervical HPV and hrHPV. On average, the collective contribution of all the selected risk factors increased (β = 1.56, 95% CI: −0.43–3.57) per quartile difference for any cervical HPV. The number of other anatomical sites with HPV had the largest positive relative contribution to any cervical HPV among FSWs (weight: 0.592). Other factors with positive relative contributions to any cervical HPV among FSWs include the total number of sexual partners (weight: 0.185), alcohol consumption (weight: 0.102), weekly number of men who pay for sex (weight: 0.080), and years of education (weight: 0.039).

The collective contribution of the selected risk factors to any high-risk cervical HPV was significantly higher compared to the contribution to any cervical HPV [β = 2.23 (95% CI: 0.72–3.75, *p* = 0.003)]. All selected risk factors showed the same relative direction (both positive and negative) to any cervical HPV and hrHPV among FSWs (Table 2).

**Figure 2:**
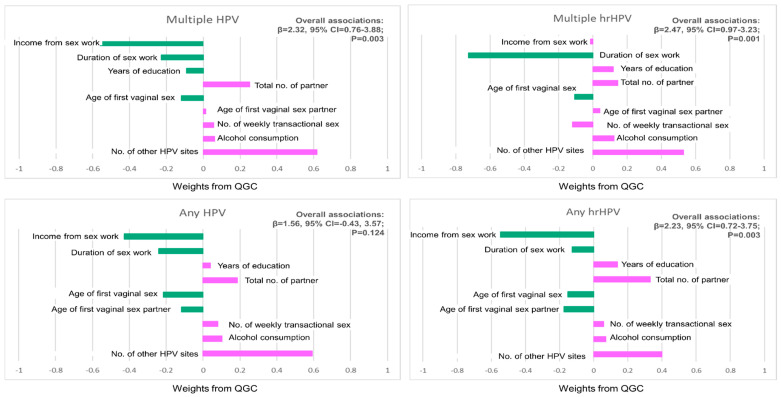
Plots of risk factor weights from QGC.

The collective contribution of all selected risk factors increased per quartile difference for multiple cervical HPV infections (β = 2.32, 95% CI: 0.76–3.88, *p* = 0.003) as well as for multiple high-risk cervical HPV infections (β = 2.47, 95% CI: 0.97–3.23, *p* = 0.001). The number of other anatomic sites with HPV infections had the largest positive relative contribution to multiple cervical HPV (weight: 0.616) and multiple high-risk cervical HPV (weight: 0.529) among FSWs. Weekly income from sex work had the highest negative relative contribution to multiple cervical HPV (weight: −0.561), while the duration of involvement in commercial sex activity had the highest negative relative contribution to multiple high-risk cervical HPV (Table 3). Figure 2 provides a graphical representation of the relative contributions of the selected risk factors for each outcome variable.

## 4. Discussion

In this study, lifestyle, behavioral, and biological risk factors collectively contributed to high-risk cervical HPV and multiple high-risk cervical HPV infections among FSWs. Specifically, the number of other anatomic sites with HPV and the total number of sexual partners showed a positive relative contribution to high-risk cervical HPV. We observed positive relationships between years of education and weekly income from sex work, as well as between age at first vaginal sex and partner’s age at first vaginal sex.

The greatest positive relative contribution of the number of other anatomical sites with HPV to high-risk cervical HPV is not unexpected, as numerous studies have shown concordance of hrHPV infections in the cervix and oral mucosa [19,20], as well as in the cervix and anal site [21,22]. A study involving 1378 women found that 27% had anal HPV infections, 29% had cervical HPV infections, and 13% had concurrent cervical–anal HPV infections [23]. Another study conducted among 1812 women reported 7.0% prevalence of oral HPV among women with cervical HPV infections, compared to 1.4% among those without cervical HPV infections [24]. In a study conducted at the First Medical School of Charles University in Prague, Czech Republic, 80% of women with oral HPV also had dual cervix–oral HPV infections [25]. The proximity of the cervical and anal anatomical sites plays a significant role in the strong relationship between HPV infections at these two sites, while the association between cervical HPV infection and oral HPV infection has been attributed to the practice of oral sex [26]. The relative contribution of HPV presence in other anatomical sites to high-risk cervical HPV is notably higher among FSWs due to their high-risk sexual activities and multiple partners.

The positive contribution of the total number of sexual partners to high-risk cervical HPV among FSWs is due to their occupation. FSWs are frequently exposed to multiple sexual partners, significantly increasing their risk of contracting HPV. This heightened exposure results in a higher prevalence of cervical HPV among FSWs compared to the general population. The frequent contact with a variety of sexual partners not only raises the likelihood of acquiring HPV but also increases the chances of encountering high-risk HPV types, such as HPV-16 and HPV-18, which are strongly associated with CC. FSWs are more prone to co-infections with multiple HPV strains, which can hinder the body’s ability to clear the virus, leading to persistent infections and a greater risk of CC development.

Alcohol consumption also had a positive relative contribution to high-risk cervical HPV infections, highlighting the role of lifestyle factors in HPV risk among FSWs. FSWs use alcohol for various reasons, often as a means of coping with the social, psychological, and environmental challenges associated with their work. Alcohol serves as a coping mechanism to manage the stress, anxiety, and emotional difficulties they encounter, such as dealing with stigma, violence, or the mental strain of transactional sex, and it also reduces inhibitions, making it easier for them to engage in sexual activities or navigate challenging interactions with clients. Some clients may also expect or encourage FSWs to drink, either to create a certain atmosphere or as part of the interaction. This consistent consumption of alcohol impairs their immune function, making it more difficult for their body to clear HPV infections and leading to persistent infections that are more likely to cause cervical abnormalities.

In our study, the finding that age at first vaginal sex has a negative relative contribution to having high-risk cervical HPV among FSWs contrasts with findings from various studies conducted on women in the general population [27,28,29], where early sexual initiation is typically associated with a higher risk of cervical HPV. This discrepancy may be explained by the unique risk profile of FSWs, where factors such as the high number of sexual partners and frequent exposure to different HPV strains may overshadow the influence of age at first sexual intercourse. In the general population, women who initiate vaginal sex at an earlier age are often exposed to HPV at a time when their immune systems may be less mature, increasing their vulnerability to persistent HPV infections [27]. However, among FSWs, the cumulative risk from multiple sexual exposures likely plays a more dominant role in determining the risk of HPV and potentially diminishes the relative impact of age at first sex.

The major strength of this study lies in the use of QGC for a comprehensive assessment of both the relative and collective contributions of selected risk factors. This approach helps us understand how different risk factors contribute individually and in a combined way to acquiring HPV infections. However, this study has limitations, including its cross-sectional design, which prevents us from determining causality or examining the timing of HPV acquisition, clearance, and persistence among FSWs.

In conclusion, our study found a significant collective contribution of lifestyle, behavioral, and biological risk factors to high-risk cervical HPV. Among these factors, the number of other anatomic sites with HPV infections, in addition to the cervix, had the greatest relative contribution, followed by the total number of sexual partners. In contrast, age at first vaginal sex showed a negative relative contribution to high-risk cervical HPV infections. Based on the findings of our study, routine screening protocols for FSWs should be enhanced to include testing for HPV infections at multiple anatomic sites. By identifying and managing infections beyond the cervix, healthcare providers can better address the overall HPV burden. Furthermore, targeted educational programs such as awareness and prevention programs should be developed specifically for FSWs to emphasize the risks associated with HPV infections at various sites and the importance of regular health check-ups and safe sexual practices.

## Figures and Tables

**Table 1:** Participants’ socio-demographic characteristics (N = 182).

Variables	Mean (SD)
Age	30.9 (6.4)
**Ethnic groups, n (%)**	
Igbo	57 (31.3)
Yoruba	14 (7.7)
Other Nigerian	111 (61.0)
Body Mass Index	27.5 (5.9)
Weekly income from sex work in Naira (USD 1—NGN 357)	10,560.4 (7295.4)
Duration of involving commercial sex activity (yr)	2.4 (2.9)
Years of completed formal education	10.2 (3.0)
Number of vaginal sex partners reported throughout life	119.3 (165.3)
Age at first vaginal sex	17.5 (2.7)
Age of first vaginal sex partner	23.5 (5.5)
Number of self-estimated weekly transactional sex	26.6 (14.2)
**Alcohol consumption (frequency of consumption of six or more units of alcohol), N (%)**	
Less than 1–2 times per month	35 (19.2)
2–4 times per month	44 (24.2)
2–3 times per week	66 (36.3)
4 or more times per week	37 (20.3)
Number of sites with HPV (other than cervix)	1.8 (0.8)

**Table 2:** Relative and combined contributions of the selected risk factors to any cervical HPV and any hrHPV from QGC.

Risk Factors	Any HPV	Any hrHPV
Income from sex work in naira	−0.427	−0.547
Duration of sex work	−0.240	−0.126
Total years of education	0.039	0.138
Total number of partners	0.185	0.331
Age at first vaginal sex	−0.215	−0.151
Age of first vaginal sex partner	−0.118	−0.175
No. of other HPV sites	0.592	0.399
No. of weekly men who pay for sex	0.080	0.058
Alcohol consumption (quantile)	0.102	0.071
β (95% CI) for combined factors	1.56 (−0.43, 3.57), *p* = 0.124	2.23 (0.72, 3.75), *p* = 0.003

Covariates: Age, ethnicity and BMI.

**Table 3:** Relative and combined contributions of the selected risk factors to multiple cervical HPV and multiple hrHPV from QGC.

Risk Factors	Multiple HPV	Multiple hrHPV
Income from sex work in naira	−0.561	−0.049
Duration of sex work	−0.227	−0.725
Total years of education	−0.091	0.119
Total number of partners	0.252	0.174
Age at first vaginal sex	−0.120	−0.107
Age of first vaginal sex partner	0.012	0.044
No. of other HPV sites	0.616	0.529
No. of weekly men who pay for sex	0.057	−0.118
Alcohol consumption (quantile)	0.061	0.133
β (95% CI) for combined factors	2.32 (0.76, 3.88), *p* = 0.003	2.47 (0.97, 3.23), *p* = 0.001

Covariates: Age, ethnicity and BMI.

## Data Availability

The datasets used and/or analyzed during the current study are available from the corresponding author upon request.

## Supplementary Materials

The following supporting information can be downloaded at: https://www.mdpi.com/article/10.3390/v17040485/s1, Table S1: *p*-values for the Spearman correlations between the selected risk factors and cervical HPV.

## Abbreviations

The following abbreviations are used in this manuscript:

BMI	Body Mass Index
CC	Cervical cancer
FSWs	Linear dichroism
HIV	Human Immunodeficiency Virus
HPV	Human papillomavirus
hrHPV	High-risk human papillomavirus
LGAs	Local Government Areas
QGC	Quantile-based G-Computation
SHINI	Sexual behavior and HPV infection in Nigerians in Ibadan
STIs	Sexually Transmitted Infections
