# Supplementary material for: Assessment of Relative Contributions of Lifestyle, Behavioral and Biological Risk Factors for Cervical Human Papillomavirus Infections in Female Sex Workers
Source: Viruses. 2025 Mar 28;17(4):485. doi: 10.3390/v17040485 (PMC12030798; doi:10.3390/v17040485)
Supplement: Supplementary file 1 [file viruses-17-00485-s001.zip › viruses-3519025-supplementary.pdf]

Supplementary Document

Supplementary Table 1: P-values for the Spearman correlations between the selected risk factors and cervical HPV

	**Income from sex work**	**Duration of sex work**	**Years of education**	**Total no. of partner**	**Age of first vaginal sex**	**Age of first vaginal sex partner**	**No. of weekly transactional sex**	**Alcohol consumption**	**No. of other HPV sites**
**Income from sex work**	< 0.001	0.39	< 0.001	0.29	0.80	0.69	< 0.001	0.39	0.62
**Duration of sex work**		< 0.001	0.68	0.44	0.17	0.32	0.69	0.66	0.93
**Years of education**			< 0.001	0.85	0.09	0.04	0.06	0.05	0.37
**Total no. of partner**				< 0.001	0.11	0.5	0.18	0.28	0.79
**Age of first vaginal sex**					< 0.001	0.01	0.66	0.25	0.38
**Age of first vaginal sex partner**						< 0.001	0.75	0.27	0.01
**No. of weekly transactional sex**							< 0.001	0.86	0.15
**Alcohol consumption**								< 0.001	0.75
**No. of other HPV sites**									< 0.001
